# Tongue microstructure physically constrains division of labor in bumblebee foraging

**DOI:** 10.1073/pnas.2527391123

**Published:** 2026-01-12

**Authors:** Zexiang Huang, Shumeng Wu, Qinglin Wu, Tianyu Mai, Jieliang Zhao, Bo Wang, Jianing Wu

**Affiliations:** ^a^School of Advanced Manufacturing, Sun Yat-Sen University, Shenzhen 518107, China; ^b^School of Chemical and Biomolecular Engineering, Georgia Institute of Technology, Atlanta, GA 30332; ^c^School of Mechanical Engineering, Beijing Institute of Technology, Beijing 100081, China; ^d^State Key Laboratory of Palaeobiology and Stratigraphy, Nanjing Institute of Geology and Palaeontology, Chinese Academy of Sciences, Nanjing 210008, China

**Keywords:** bumblebee, nectar feeding, division of labor, allometry, liquid entrainment

## Abstract

Bumblebee (*Bombus terrestris*) is a primitively eusocial species that sustains its colonies by dividing foraging duties between queens and workers. Previous research emphasizes hormonal, metabolic, and behavioral contrasts between the castes but seldom considers why queens relinquish foraging once workers emerge. Here, we link foraging efficiency to tongue microstructure, providing physical evidence for the shift in bumblebee foraging behavior.

Understanding how social-insect castes allocate labor has long been central to behavioral ecology, especially regarding how caste-specific morphological and behavioral differences translate into variation in foraging performance (1). Bumblebee (*Bombus terrestris*) is an influential model because it straddles the transition from primitively to highly eusocial life histories while remaining amenable to both field and laboratory study (2). In bumblebee species, adult queens are always larger than workers, but show virtually no external morphological differences (3). However, queens forage intensively only during nest founding and cease once workers take over, whereas workers continue to collect nectar throughout the season (4). Existing evidence attributes this caste divergence to endocrine regulation, energy metabolism, and task specialization, but no study has yet examined whether tongue microstructure constrains foraging efficiency (5–7). Here, we integrate postmortem scanning electron microscopy (SEM) with in vivo high-speed filming to establish a form–function relationship of the bee tongue, providing morphological and physical evidence for queen-worker divergence. We show that, although hair spacing increases more slowly than tongue length under allometric growth, their positive scaling still renders the tongues of large queens excessively porous, limiting nectar retention and making queens less efficient nectar foragers than workers.

To sustain efficient nectar intake, bumblebees have evolved an elongated proboscis made up of a pair of galeae and labial palpi, which form a feeding tube surrounding a hairy tongue (Fig. 1*A*). The tongue, or glossa, partitioned into segments by transverse cuticular rings, each decorated by slender hairs, is the primary feeding structure for extracting nectar from the floral tube. We dissected 99 *B. terrestris* adults (*n*_worker_ = 67 and *n*_queen_ = 32; body mass *M* = 65 to 810 mg) to examine their tongue morphology (*SI Appendix*). Here, we report two critical parameter that capture tongue architecture at distinct scales, namely the macroscale measure of tongue length *L*_T_ and the microscale measure of hair spacing *L*. As shown in Fig. 1*C*, Log-log regressions showed near-isometric scaling of *L*_T_ but shallow allometric scaling of *L*. Kernel-density ridges show queens cluster at higher body mass with longer tongues and wider hair spacing. We hypothesize that these geometric differences underpin the caste-specific division of labor during nectar collection.

**Fig. 1.**
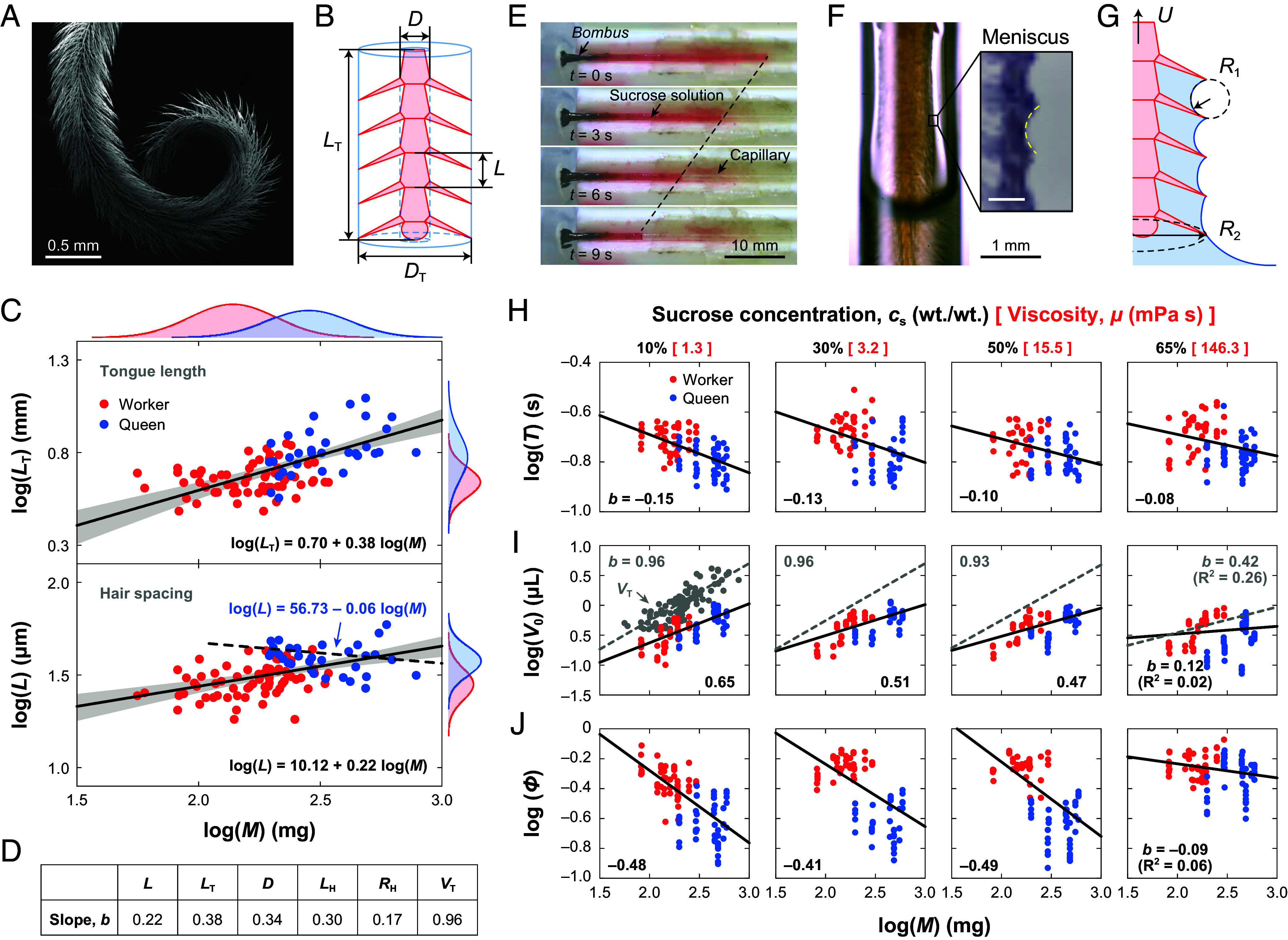
Tongue morphology and feeding performance. (*A*) SEM image of a queen bumblebee tongue. (*B*) Schematic of tongue geometry. Blue profile outlines the usable nectar volume *V*_T_. (*C*) Log-log allometry of *L*_T_ and *L* versus body mass. Black lines give the least-squares fits and the gray bands denote their 95% CI. The dashed line indicates the queen-only regression, indicating that hair spacing is nearly size-invariant among queens. Kernel-density ridges depict lognormal distributions for workers (red) and queens (blue). (*D*) Table summarizing the allometric exponents for tongue geometry. (*E*) Time-lapse imaging and (*F*) High-speed filming of feeding behavior (*c*_s_ = 50% wt./wt.). The *Inset* shows the curved meniscus between hairs, (Scale bar: 20 µm.) (*G*) Schematic of tongue withdrawal illustrating retraction speed *U* and meniscus radii *R*_1_ and *R*_2_, whose curvature difference generates Laplace pressure that entrains nectar. (*H*) Lapping time *T*, (*I*) Per-lap volume *V*_0_, and (*J*) Filling factor *Φ* versus body mass across sucrose concentrations (or viscosities). For each subpanel, the regression line is the least-squares fit to the model log(*Y*) = *a* + *b* log(*M*), where *b* is the fitted slope reported in each panel. Gray points in (*I*) show theoretical capacity *V*_T_. For clarity, in the 30%, 50%, and 65% panels, only the gray fit line is shown.

Lapping, the common nectar-feeding mechanism of bumblebees, involves back-and-forth motion of the tongue to collect nectar (8). To test how hair spacing influences nectar uptake, we recorded videos (*n*_video_ = 3 to 6 per bee) of bumblebees (*n*_worker_ = 13 and *n*_queen_ = 9) capturing artificial nectars with different sugar contents, namely different viscosities (Movie S1). Tracking the liquid–air meniscus inside the capillary allowed us to measure the liquid intake volume per lap *V*_0_ and lapping time *T* of each bee, and compute the ingestion rate *Q* (Fig. 1*E*). Across three orders of magnitude in viscosity, lapping time was nearly constant in both castes and varied only weakly with body mass (*T* ~ *M*^−0.1^; Fig. 1*H*), indicating that bees have the ability to adjust retraction force in response to nectar viscosity (8). Queens lapped slightly faster than workers, but resultant differences in retraction speed (*U* = *L*_T_/*T*) were minor compared to those driven by tongue geometry.

Previous work assumed that the entire space inside the outer envelope of the tongue hairs fills completely with liquid per lap (8, 9). We modeled this space as a cylindrical sleeve with negligible hair volume and defined theoretical nectar-holding capacity as *V*_T_ = *π*(*D*_T_^2^ − *D*^2^)*L*_T_/4, where *D*_T_ is outer diameter of the tongue considering hairs, and *D* is the diameter of tongue base (Fig. 1*B*). Across individuals, *V*_T_ scaled approximately in direct proportion to body mass. However, the measured per-lap volume *V*_0_ increased with size more slowly than *V*_T_ (Fig. 1 *D* and *I*). Given that lapping time varied little across body sizes (Fig. 1*H*), the ingestion rate *Q* = *V*_0_/*T* exhibited the same subisometric scaling as *V*_0_. This aligns our laboratory results with field observations showing that larger workers achieve greater absolute nectar loads and intake rates, but that these gains do not increase proportionally with body size (10, 11).

To account for differences in tongue size, we normalized the observed intake volume *V*_0_ by *V*_T_ and defined a filling factor *Φ* = *V*_0_/*V*_T_. We found that *Φ* declined with body mass across viscosities, indicating that size-related differences in nectar retention. In addition, when individuals were matched for body mass or tongue length, queens consistently exhibited lower *Φ* than workers (Fig. 1*J*). This difference arises because queens consistently possess wider hair spacing than workers at a given body size (Fig. 1*C*). Given that each colony provided only a single queen, who was invariably the largest individual (7), queens consistently represented the upper extreme of hair-spacing variation within colonies, thereby exhibiting size-independent hair spacing within queens. Overall, the reduced filling efficiency in queens compared to equally sized workers, along with their consistently wider hair spacing, demonstrates that differences in tongue microstructure drive variation in nectar-uptake performance. Consequently, queens cannot fully exploit the internal tongue volume to capture nectar, which may make them less suited to foraging and promote caste-based division of labor.

Previous theory typically focused on viscous entrainment by the hairy structures, overlooking how the same microstructure provides additional support for the liquid (8, 12, 13). High-speed films recorded a curved meniscus spanning the gaps between hairs during tongue withdrawal, whose curvature imposes additional negative Laplace pressure on the liquid surface (14) (Fig. 1*F* and Movie S2). Thus, capillary pressure provides a driving force for entrainment here rather than resistance. The result is a positive pressure difference along the tongue, Δ*P*_c_ ≈ 2*γ*/*L*, where *γ* is surface tension (Fig. 1*G* and *SI Appendix*). Accordingly, increasing hair spacing weakens the supportive pressure generated by the liquid bridge, especially in dilute nectar. Our statistical results show that hair spacing increases with body mass with an exponent of ~0.22, below isometry and shallower than the scaling of tongue length *L*_T_ (Fig. 1*C*). This underscaling reflects a compromise that partly, though not fully, optimizes microgeometry as body size increases.

To capture the coeffects of viscosity and capillarity, we propose a characteristic length *L*_C_ = (*LL*_T_)^1/2^, which merges the macro- and microscale of hairy tongue and clearly separates the castes (*SI Appendix*). Workers have small *L*_C_ that keeps them in the capillary regime, making nectar uptake largely insensitive to viscosity and enabling flexible foraging across nectar types. Queens, with much larger *L*_C_, sit closer to the viscous regime and therefore perform better with high-viscosity nectar (Fig. 2*A*). We farther map the data into a dimensionless space with Bond number *Bo* = *ρgL*_C_^2^/*γ* and Capillary number *Ca* = *μU*/*γ* = 2*μL*_T_*f/γ*, revealing clear differences between queens and workers. Specifically, constant *Φ* corresponds to steep isolines in this *Bo*-*Ca* space (*Ca* ~ *Bo*^5^), indicating that maintaining high nectar retention requires rapidly increasing viscous entrainment at larger scales. However, morphology imposes a much shallower scaling (*Ca* ~ *Bo*^2/3^). Consequently, as body size increases, bees cannot sustain the required steep *Bo*-*Ca* relationship to maintain high filling factor (Fig. 2*B* and *SI Appendix*). This mismatch between physical demands and morphological constraints explains why queens become less efficient nectar foragers.

**Fig. 2.**
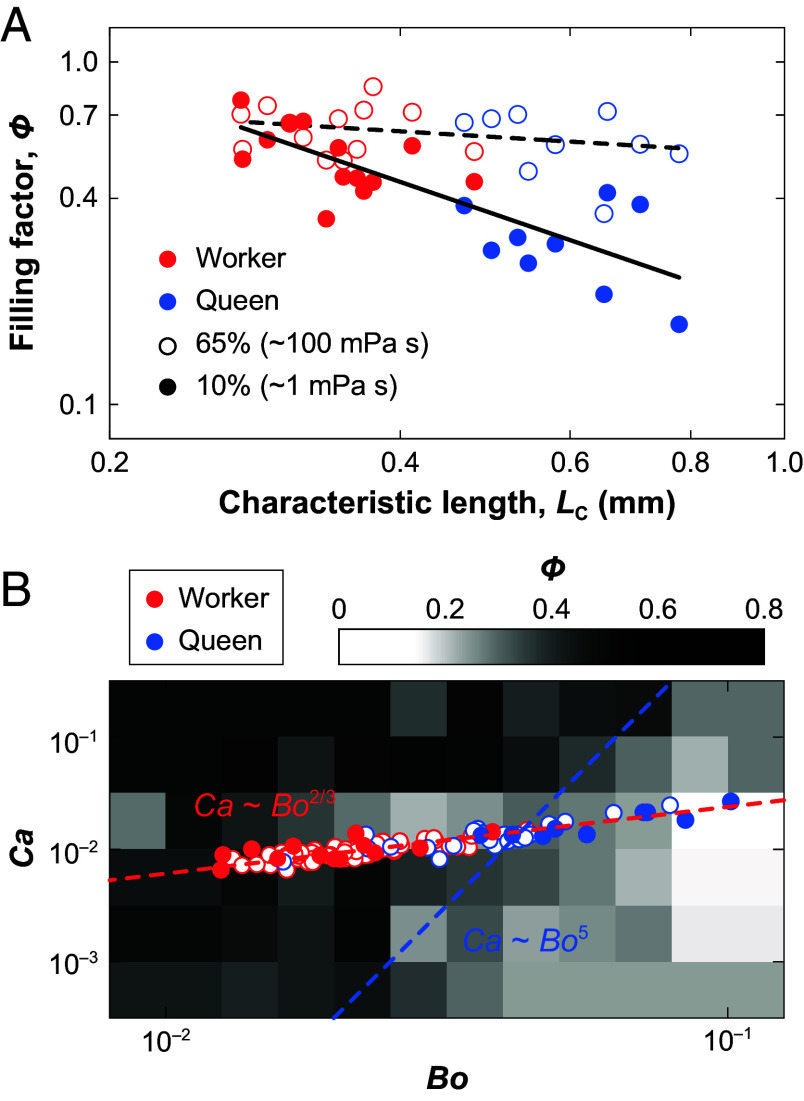
Theoretical framework for nectar uptake. (*A*) Relation between filling factor *Φ* and characteristic length *L*_C_. One point per bee with the highest *Φ* across trials used as its uptake capacity. The solid line (10 % sucrose, slope = −0.96, R^2^ = 0.65) and dashed line (65% sucrose, slope = −0.17, R^2^ = 0.09) are least-squares fits. (*B*) Dimensionless *Bo*-*Ca* framework with *Φ* encoded in grayscale. Open points denote all individuals in the morphological dataset and filled points mark the subset with feeding trials. The red dashed line shows the morphology-constrained trajectory at 50% sucrose. The blue dashed line approximates *Φ* ≈ 0.5 isoline.

In summary, our integrative morphological and kinematic analysis reveals that a scaling mismatch between tongue length and hair spacing in bumblebee queens limits their nectar capture. This physical constraint offers a mechanistic explanation for why queens relinquish foraging once workers emerge, underscoring how subtle deviations in microstructure can influence the division of labor in social insects. Our work offers a simple framework for pairing individual-level structure and function to predict performance. Beyond bumblebees, it can be extended across bee taxa by shifting the bee-flower problem from purely geometric matching (tongue length versus corolla depth) to physical matching between tongue porosity and nectar properties (viscosity, surface tension). This framework could apply to other hairy fluid-capture systems and inform bio-inspired porous interfaces.

## Material and Methods

The detailed methods are described in *SI Appendix*. Briefly, *B. terrestris* queens and workers were offered sucrose solution in a glass capillary, while their feeding behaviors were recorded. The structural parameters of their tongues were measured from the SEM images. For the theoretical analysis, a *Bo*-*Ca* framework was used to link tongue structure and function.

## Supplementary Material

Appendix 01 (PDF)

Dataset S01 (XLSX)

Movie S1. Feeding behavior of a queen bumblebee with a 50% (wt./wt.) sugar solution.

Movie S2. High-speed video of a queen bumblebee feeding from a glass capillary containing 50% (wt./wt.) sucrose solution.

## Data Availability

Study data are included in the article and/or supporting information.

## References

[1] C. R. Smith, A. L. Toth, A. V. Suarez, G. E. Robinson, Genetic and genomic analyses of the division of labour in insect societies. Nat. Rev. Genet. **9**, 735–748 (2008). 18802413 10.1038/nrg2429

[2] G. Ghisbain, L. Chittka, D. Michez, Bumblebees. Curr. Biol. **35**, R206–R211 (2025). 40132549 10.1016/j.cub.2025.01.041

[3] J. J. M. Pereboom, W. C. Jordan, S. Sumner, R. L. Hammond, A. F. G. Bourke, Differential gene expression in queen–worker caste determination in bumble-bees. Proc. R. Soc. B. Biol. Sci. **272**, 1145–1152 (2005). 10.1098/rspb.2005.3060 PMC1559810 16024376

[4] D. Goulson, Bumblebees: Behaviour, Ecology, and Conservation (Oxford University Press, 2010).

[5] C. Grüter, C. Menezes, V. L. Imperatriz-Fonseca, F. L. W. Ratnieks, A morphologically specialized soldier caste improves colony defense in a neotropical eusocial bee. Proc. Natl. Acad. Sci. U.S.A. **109**, 1182–1186 (2012). 22232688 10.1073/pnas.1113398109 PMC3268333

[6] K. M. Kapheim, Nutritional, endocrine, and social influences on reproductive physiology at the origins of social behavior. Curr. Opin. Insect Sci. **22**, 62–70 (2017). 28805640 10.1016/j.cois.2017.05.018

[7] M. Ayasse, S. Jarau, Chemical ecology of bumble bees. Annu. Rev. Entomol. **59**, 299–319 (2014). 24160431 10.1146/annurev-ento-011613-161949

[8] A. Lechantre , Essential role of papillae flexibility in nectar capture by bees. Proc. Natl. Acad. Sci. U.S.A. **118**, e2025513118 (2021). 33931548 10.1073/pnas.2025513118 PMC8126835

[9] L. D. Harder, Effects of nectar concentration and flower depth on flower handling efficiency of bumble bees. Oecologia **69**, 309–315 (1986). 28311376 10.1007/BF00377639

[10] D. Goulson , Can alloethism in workers of the bumblebee, *bombus terrestris*, be explained in terms of foraging efficiency? Anim. Behav. **64**, 123–130 (2002).

[11] J. Spaethe, A. Weidenmüller, Size variation and foraging rate in bumblebees (*Bombus terrestris*). Insectes Soc. **49**, 142–146 (2002).

[12] W. Kim, T. Gilet, J. W. M. Bush, Optimal concentrations in nectar feeding. Proc. Natl. Acad. Sci. U.S.A. **108**, 16618–16621 (2011). 21949358 10.1073/pnas.1108642108 PMC3189050

[13] A. Nasto, P.-T. Brun, A. E. Hosoi, Viscous entrainment on hairy surfaces. Phys. Rev. Fluids **3**, 24002 (2018).

[14] Z. Cheng , Viscous-capillary entrainment on bioinspired millimetric structure for sustained liquid transfer. Sci. Adv. **9**, eadi5990 (2023). 37682994 10.1126/sciadv.adi5990 PMC10491213

