# Supplementary material for: Tongue microstructure physically constrains division of labor in bumblebee foraging
Source: Proc Natl Acad Sci U S A. 2026 Jan 12;123(3):e2527391123. doi: 10.1073/pnas.2527391123 (PMC12818393; doi:10.1073/pnas.2527391123)
Supplement: Supplementary file 1 — Appendix 01 (PDF) [file pnas.2527391123.sapp.pdf]

Supplemental Information

Tongue microstructure physically constrains division of labor in bumblebee foraging

Zexiang Huang, Shumeng Wu, Qinglin Wu, Tianyu Mai, Jieliang Zhao, Bo Wang, Jianing Wu

Bumblebees

Totally 99 *Bombus terrestris* (n_{worker} = 67 and n_{queen} = 32; body mass 65–810 mg) from the Biobest firm were used for the in *vivo* experiments. The colony was kept at the temperature of 25°C and humidity of 65 to 70% under a 12 h L:12 h D photoperiod. They were fed every 2 d with pollen candies, and a 1 mm diameter capillary tube filled with sucrose solution imitating the nectar is provided to them, and the whole process was recorded in 60fps (for liquid uptake measurement) and 600fps (for microstructure and fluid mechanics). Worker and queen bees that had died naturally in the colony were collected within 12 hours and stored at 4°C until dissection.

Tongue Geometry

Each sample was weighed on a microbalance before dissection. The tongue was then photographed under a stereomicroscope, providing a macroscopic image from which glossal length was later extracted by digital measurement. Immediately afterwards the tongue was fixed in 2.5 % glutaraldehyde, dehydrated through a graded ethanol series and sputter coated with 10 nm Pd for scanning electron microscopy. We measured the hair spacing L, tongue rod diameter D, hair length L_H and hair radius R_H from SEM image.

Artificial Nectar

Since sugar (basically sucrose, glucose, and fructose) in nectar is the most essential energy source for honey bee's flight, thermoregulation, and hence and development, and nectar quality is commonly indicated by its sugar concentration, and hence we used sucrose solution as artificial nectar for experiments. The artificial nectar consisted of sucrose solution at three concentrations (10%, 30%, 50% and 65% wt./wt.). For sucrose solution, the variations of the viscosity as a function of the sucrose concentrations at 30°C can be expressed as

$$\mu(x) = (1097)^{-1} 10^{0.8752x/(1-x)} 10^{1.01c^2}. \quad (1)$$

where $x = c/100$ is the sucrose concentration (wt%) and μ is the viscosity in Pa s. The variation of the density of various sugar solutions as a function of their viscosity can be expressed as

$$\rho = 1134\left[\ln\left(\frac{\mu + 0.00132}{0.00175}\right)\right]^{1/10}. \quad (2)$$

The surface tension γ of the nectar has been found to be independent of sugar concentration, with a constant value around 0.074 N/m. However, it is very hard to have the nominal γ, because γ decreases as soon as there are some impurities and we use $\gamma = 0.05$ N/m in the study as a conservative estimate.

Tongue Kinematics

Before beginning observation of the drinking process, bumblebees were starved at the room temperature of 26°C in the dark from 2 to 4 h. A bumblebee was then transferred into a centrifuge tube of 15 mL with a 4-mm hole at the tip. After a habituation phase of 3

min, the extension of the proboscis was motivated by presenting a drop of a solution of diluted honey. Finally, a capillary tube with an inner diameter D_c = 1 mm containing a sweet solution of known sugar concentration was presented to the bumblebee. The inner diameter is similar to the nectar tube width of some plants visited by bumblebees. Two complementary imaging systems captured tongue kinematics. A camera at 60 fps captured the overall advance of the liquid–air meniscus in the capillary, providing the macroscopic displacement trace used to compute ingestion rate (Movie S1). A high-speed camera mounted on a 10× long-working-distance objective and running at 600 fps resolved local hair deployment and meniscus curvature (Movie S2).

Intake Volume and Lapping Frequency

The position p(t) of the meniscus at the liquid–air interface was measured as a function of time, and the ingestion rate was obtained from $Q(t) = \pi D_c^2 v_m(t)/4$, where $v_m(t) = dp(t)/dt$ is the velocity of the meniscus. Since $p(t) \sim t$, v_m and Q are constant, the periodic advance/recoil oscillations of p(t) were isolated with a high‐pass Butterworth filter. Analysis of the curve gives access to details about the dynamical process such as the volume V_0 of fluid captured per lap and the lapping time T_L (lapping frequency $f = 1/T_L$).

Filling Factor

The usable tongue volume V_T should account for the partial hair erection at the moment of withdrawal. Treating each papilla as a slender elastic rod acted on by a linear viscous drag gives

$$d = d_m\left(1 - e^{-11T_R/T}\right), \quad T = 1.42\frac{\mu}{E}\left(\frac{R_H}{L_H}\right)^4. \tag{3}$$

Here $d_m = L_H\sin55°$ is the maximum papilla deflection (fully open), $T_R = T_L/2$ is the measured retraction time, E = 1.1 MPa is Young's modulus of hairs. Therefore, the effective cavity volume of the hairy sleeve is $V_T = \pi(D_T^2 - D^2)L_T/4$, where $D_T = D + 2d$ is the distance between the tips of the tongue hairs. Finally, the filling factor used to normalize intake across castes and nectar types becomes

$$\Phi = \frac{V_0}{V_\mathrm{T}} = \frac{4V_0}{\pi[(D+2d) - D^2]L_\mathrm{T}}. \quad (4)$$

For each bee and for each sucrose concentration, we performed 3–6 repeats and defined Φ_{max} as the maximum Φ across those repeats at that concentration.

Physical Scaling and Characteristic Length

High-speed films (600 frames/s) recorded a curved meniscus spanning the gaps between hairs as the tongue withdrew, whose curvature imposes an additional negative Laplace pressure on the liquid surface. Thus, the capillary pressure provides a driving force for entrainment here rather than resistance. The result is a positive pressure difference along the tongue, $\Delta P_c = P_1 - P_2 = \gamma(1/R_1 - 1/R_2)$, where γ is surface tension, R_1 and R_2 are the radius of curvature of the meniscus between hairs and the meniscus at the entrainment transition zone, respectively. With $R_1 \approx L/2 \sim 10$ μm and $R_2 \approx D_T/2 \sim 100$ μm, the term $1/R_2$ is an order of magnitude smaller than $1/R_1$ and can be neglected, so $\Delta P_c \approx 2\gamma/L$.

Balancing this capillary pressure $\Delta P_c \approx 2\gamma/L$ against the hydrostatic pressure $\Delta P_c \approx \rho g L_T$ gives a simple geometric prediction $L \sim 2\gamma/(\rho g L_T)$. Hence effective capillary retention demands that hair spacing decline in inverse proportion to tongue length, $L \sim L_T^{-1}$. Hence effective capillary retention demands that hair spacing decline in inverse proportion to

tongue length, $L \sim L_T^{-1}$. If tongue length scales isometrically with body mass, $L_T \sim M^{1/3}$, the prediction becomes $L \sim M^{-1/3}$. Our statistical results show a much shallower slope of 0.22, far from the ideal −1/3, yet still below isometry. The view from viscous entrainment leads to the same qualitative conclusion. The volume retained per lap results from competition between the tongue retraction speed U and the drainage speed $U_{drain} = \rho g k/\mu$, where μ is viscosity and k is permeability. Widening the hair spacing increases the permeability and therefore promotes drainage, especially in low-viscosity nectar. To reveal the co-effects of viscosity and capillarity, we propose a characteristic length $L_C = (LL_T)^{1/2}$, which merges the macro- and micro- length scale of hairy tongue.

Bo–Ca Framework for Nectar Feeding

Physically, maintaining a constant Φ requires balancing viscous entrainment of nectar onto the tongue against gravitational drainage off the tongue. This balance is captured by two dimensionless numbers: Bond number $Bo = \rho g L_C^2/\gamma$ quantifies the influence of gravity relative to capillarity and the Capillary number $Ca = \mu U/\gamma = 2\mu L_T f/\gamma$ expresses the balance between viscous and surface-tension forces. Thus, constant Φ contours follow a power-law relationship, $Ca \sim Bo^n$, where the exponent n reflects how sensitively viscous uptake must scale to counteract gravity. In the capillarity-dominated limit (very low Bo), a classical Landau–Levich film forms on the tongue with thickness h scaling as $Ca^{2/3}$. As Bo grows and gravity begins to contribute, the scaling of retained nectar shifts toward a shallower $h \sim Ca^{1/2}$ (the Derjaguin gravity regime). A constant fill factor essentially fixes h (as a fraction of tongue hair length), so increasing Bo demands a rapid rise in Ca to preserve Φ. In fact, our analysis indicates that for high filling efficiency ($\Phi \approx 0.5$), Ca would

have to increase on the order of Bo^5 to compensate - a very steep trajectory that highlights the strong influence of gravity at larger scales. In other words, beyond a certain size, even slight increases in Bo (due to larger tongue dimensions) would require disproportionately large increases in viscous uptake to maintain the same Φ, underscoring why high-Φ isolines have such steep slopes in theory.

However, the empirical morphological relationships observed in bumblebees impose a much shallower trajectory. Morphologically, L_C scales sub-linearly with tongue length ($L_C \sim L_T^{0.75}$), resulting in $Bo \sim L_C^2 \sim L_T^{1.5}$ and Ca $\sim L_T$ (assuming nearly constant lapping frequency f). Thus, empirical morphology predicts $Ca \sim Bo^{2/3}$, significantly shallower than the theoretical requirement ($Ca \sim Bo^5$). This mismatch implies that bees are structurally unable to match the steep scaling necessary to maintain high filling factors at larger scales. Consequently, larger bees, particularly queens, inevitably operate at lower Φ values due to their relatively coarser tongue structures.

We analyzed these relationships at a representative nectar viscosity corresponding to approximately 50% sucrose (~15 mPa·s), near the optimal nectar concentration typically found in nature. Changing nectar concentration (viscosity μ) would shift the bee's position vertically in the Ca–Bo plane without altering the fundamental scaling mismatch and resulting structural limitations.

Movie S1 (separate file). Feeding behavior of a queen bumblebee with a 50% (wt./wt.) sugar solution.

Movie S2 (separate file). High-speed video of a queen bumblebee feeding from a glass capillary containing 50% (wt./wt.) sucrose solution.
